# Novel Techniques for Mapping DNA Damage and Repair in the Brain

**DOI:** 10.3390/ijms25137021

**Published:** 2024-06-27

**Authors:** Jenna Hedlich-Dwyer, Joanne S. Allard, Veronica E. Mulgrave, Glen E. Kisby, Jacob Raber, Natalie R. Gassman

**Affiliations:** 1 Department of Pharmacology and Toxicology, Heersink School of Medicine, The University of Alabama at Birmingham, Birmingham, AL 35294, USA; jhedlich@uab.edu; 2 Department of Physiology & Biophysics, Howard University College of Medicine, Washington, DC 20059, USA; joanne.allard@howard.edu (J.S.A.); vmulgrave@desu.edu (V.E.M.); 3 Department of Biomedical Sciences, College of Osteopathic Medicine, Western University of Health Sciences, Lebanon, OR 97355, USA; gkisby@westernu.edu; 4 Department of Behavioral Neuroscience, Neurology, and Radiation Medicine, Division of Neuroscience, ONPRC, Oregon Health & Science University, Portland, OR 97239, USA; raberj@ohsu.edu

**Keywords:** DNA damage, DNA damage response, DNA repair, neurodegeneration, neurological disease, fluorescent microscopy, DNA adduct, immunohistochemistry, mitochondria, spatial mapping techniques

## Abstract

DNA damage in the brain is influenced by endogenous processes and metabolism along with exogenous exposures. Accumulation of DNA damage in the brain can contribute to various neurological disorders, including neurodegenerative diseases and neuropsychiatric disorders. Traditional methods for assessing DNA damage in the brain, such as immunohistochemistry and mass spectrometry, have provided valuable insights but are limited by their inability to map specific DNA adducts and regional distributions within the brain or genome. Recent advancements in DNA damage detection methods offer new opportunities to address these limitations and further our understanding of DNA damage and repair in the brain. Here, we review emerging techniques offering more precise and sensitive ways to detect and quantify DNA lesions in the brain or neural cells. We highlight the advancements and applications of these techniques and discuss their potential for determining the role of DNA damage in neurological disease.

## 1. Introduction

DNA is continuously exposed to endogenous metabolites, exogenous chemicals, and toxins, creating diverse DNA lesions and strand breaks. Detection and repair of this DNA damage is essential to maintaining genomic stability, and cells have developed complex and highly regulated DNA damage signaling and repair pathways. In the brain, these pathways take on increased importance due to the high transcriptional activity and energetics of post-mitotic neurons, which comprise a significant portion of the central nervous system. These long-lived cells are vulnerable to exogenous exposures stemming from our lifestyle behaviors and environments and susceptible to elevated reactive species from endogenous metabolic processes.

Importantly, some forms of DNA damage in neurons play essential physiological roles in memory and learning. Inducing neuronal activity increases double-strand breaks (DSBs) and leads to the upregulation of immediate early genes (IEGs) while increasing DSBs and inhibiting DSB repair impair long-term memory and altering IEG expression [1,2,3,4,5,6,7].

Beyond the physiological role of DNA damage within neurons, DNA lesions and strand breaks must be dealt with quickly and effectively in neurons as well as other neural cell types, i.e., astrocytes, oligodendrocytes, and microglia, to avoid triggering senescence and cell death. The accumulation of DNA damage, particularly oxidative lesions, single-strand breaks (SSBs) and DSBs, is strongly associated with aging and neurodegeneration [8,9].

While the associations between elevated DNA damage and reduced DNA repair in neurodegeneration and neuropsychiatric diseases are well documented, numerous DNA damage response (DDR) mechanisms drive these changes, including alterations in DNA repair pathways, changes in chromatin structure, and post-translational modifications regulating chromatin, DNA repair, and protein degradation pathways [10,11,12,13]. These highly critical processes play critical and often interrelated roles in maintaining genomic integrity, and alterations in their function promote the accumulation of DNA damage, genomic rearrangements, and mutations. These outcomes compromise neural cell function, induce apoptosis, or promote increased cell proliferation and growth [1,14,15]. As a result, the accumulation of DNA damage within the brain is associated with a number of pathologies, including those triggered by traumatic brain injuries and cerebral ischemic strokes [16,17,18].

Neurological disorders, including cerebrovascular events, neurodegenerative, and trauma, have some of the highest rates of morbidity, disability, and mortality globally [19]. In 2019, neurological disorders were responsible for nearly 10 million deaths and 349 disability-adjusted life-years (DALYs) lost globally [20,21]. The increasing burden of neurological disorders stemming from population increases, growing life expectancy, urbanization and environmental exposures is increasing the healthcare and economic burden globally [22]. Therefore, understanding the etiology of these disorders and developing improved diagnostics and therapeutics is increasingly critical.

The progressive accumulation of DNA damage is a common feature of these disorders and aging [23,24,25,26,27]. Therefore, mapping and measuring DNA damage within the brain may offer tools for early diagnosis, assessment of progression, and evaluation of the effectiveness of drug therapies. Of note, there is increasing evidence that elevated levels of DNA damage, including oxidative lesions, contribute significantly to cognitive impairment in major psychiatric disorders like depression, bipolar disorder, and schizophrenia [28,29,30,31,32,33]. Post-contextual fear training administration of Amifostine (WR-2721), which reduces DSBs, affects long-term contextual fear memory, while administrations of etoposide, which increases DSBs, affects contextual and cued fear memory [34]. Amifostine also mitigates cognitive injury induced by simulated space irradiation in male, but not female, mice [35]. These findings demonstrate DNA damage’s complex role in the brain and the importance of measuring changes in DNA lesion types and amounts within specific brain regions.

Several comprehensive reviews have been published discussing the role of DNA damage in neurodegenerative disease and neuropsychiatric disorders (see [8,28]). However, our inability to precisely analyze the DNA damage and map specific DNA damage lesion types with anatomical- or cell-specific accuracy has hampered our understanding of the extent and persistence of DNA damage in the brain and its role in neurodegeneration and neurodevelopment. The most commonly used methods and techniques to assess DNA damage in the brain focus on direct measurement of the oxidative lesion 8-oxo-7,8-dihydroguanine (8-oxoG) or indirect measurements of strand breaks using phosphorylated H2AX (γH2AX). While these methods have been used for decades to provide critical insight into DNA damage in the brain, they limit a more detailed assessment of the role environmental agents and other inducers of DNA lesions play in aging or neurological disorders.

Significant advancements in DNA damage detection methods have occurred over the past ten years, accelerating our knowledge of DNA damage in numerous tissues, including the brain. Here, we briefly review the emerging DNA damage detection methods that have offered new insight into DNA damage and repair in the brain. Further, we offer a perspective on new avenues to advance our understanding of DNA damage in the brain and its potential as an early indicator of neurological disorders.

## 2. DNA Damage Detection Strategies

### 2.1. Existing Strategies for the Detection of DNA Lesions within the Brain

DNA damage characterization within the brain is most frequently carried out by immunoblotting, immunohistochemical, or immunofluorescent staining for 8-oxoG and γH2AX (Table 1). Numerous reports have demonstrated elevated levels of oxidative DNA damage and DNA strand breaks within brain sections of rodents and humans. While these examples are not exhaustive, elevated brain levels of γH2AX are observed in aging [36,37], Alzheimer’s disease [38,39,40], Parkinson’s disease [41,42], Huntington’s disease [43,44], excitoneurotoxicity and seizures [45,46], and in healthy brains in response to activities that stimulate learning and memory [1,3]. Elevated levels of γH2AX have also been detected in peripheral blood mononuclear cells (PBMC) of patients with Huntington’s disease [47]. When combined with measurements of p53-binding protein 1 (53BP1), an additional marker for double-strand breaks in the brain, these markers can discriminate the repair of DSBs by homologous recombination (HR) and non-homologous end-joining (NHEJ) [41,48,49].

**Table 1:** Summary of detection strategies for assessing DNA damage in the brain.

Methods	Advantages	Limitations	Refs
***INDIRECT***			
Immunoblotting	- Multiple DDR and DNA repair proteins - Compatible with surrogate blood markers	- Specificity of antibodies for protein targets	[41,48,49,50,51,52,53]
Immunohistochemical	- DDR and DNA repair proteins - Compatible with surrogate tissues or cells	- Specificity of antibodies for targets of interest - Differences in tissue processing methods for detection - Surrogate markers may not reflect 1:1 events in the brain	[1,3,36,37,38,39,40,41,42,43,44,45,46,48,49]
ChIP sequencing methods	- Robust capture methods and established techniques - Compatible with DDR proteins or specific DNA repair proteins or pathways	- Position of lesions less precise - Need for high-specificity antibodies - Need for adequate control for evaluating DNA versus DNA damage interactions - Surrogate markers may not reflect 1:1 events in the brain - Significant input of material and limits of detection for low-prevalence events	[1,2]
Fluorescent reporters	- Assessment of DNA repair capacity - Exploit specific repair mechanism (PRISM)	- Artificially induces DNA damage (I-SceI) - Post-event monitoring for genotoxic exposures	[54,55,56,57,58]
PCR	- Mitochondrial DNA damage assessment - Compatible with surrogate blood markers	- Specific DNA lesions cannot be identified - Surrogate markers may not reflect 1:1 events in the brain	[59,60,61,62,63,64,65,66,67,68,69,70,71]
***DIRECT***			
Immunohistochemical/ Immunofluorescence	- Identify specific lesions	- Specificity of antibodies for DNA lesions - Unwinding of DNA to detect lesions in situ	[61,72,73,74,75,76,77,78,79,80,81,82,83,84]
Mass Spectrometry	- Larger number of DNA lesions detected from oxidative to acrolein	- A significant amount of brain tissue for DNA isolation - Need for isotopic standards	[78,85,86,87,88,89,90]
Comet Assay	- Strand breaks and abasic sites - Ability for single nucleoid analysis - Specific DNA lesions are detected when combined with DNA repair enzymes or treatment strategies	- Mixture of lesions detected - Crosslinks and DNA protein crosslinks need specific protocols for detection - Not compatible with formalin-fixed samples	[87,88,91,92,93,94,95,96]
HPLC	- Lesion detection within genomic DNA - Lesion detection in mitochondrial DNA with specific isolation	- Significant amount of brain tissue genomic or mitochondrial DNA - May require specialty columns or enhanced separation methods - May require standards	[78,97,98,99]
Adapter or lesion-specific sequencing techniques	- Robust capture strategies using click or biotin chemistry - Specific labeling of DNA lesion targets through end breaks, lesion sites, or synthesis -Modifiable and adaptable protocols for analysis integration with single cell or other approaches	- Need for high specific antibodies for specific DNA lesions -Enzyme-mediated methods detect lesion classes based on specific enzymes used -Variable amount of DNA may be needed based on the desired analysis method	[100]
Enzyme-mediated labeling or sequencing strategies	- DNA repair enzymes allow specific lesion class detection -Modifiable and adaptable protocols compatible with spatial imaging or transcriptomics - Compatible with isolated DNA, cells, or frozen or formalin-fixed tissues - Single nuclei analysis possible	- Lesion classes may be large depending on the enzymes used - Optimization for specific tissue and cell types may be required	[101,102]

Direct lesion detection through immunoassay, enzymatic labeling, or mass spectrometry is also conducted. Elevated abasic sites have been detected within brain tissues and isolated DNA using an aldehyde reactive probe (ARP) in Parkinson’s disease, amyotrophic lateral sclerosis (ALS), and after ischemic/reperfusion injuries [61,72,73]. Elevated abasic sites were frequently detected in conjunction with increased levels of 8-oxoG, a more commonly quantified marker [72,73]. Elevated 8-oxoG levels are observed in the brain and other tissues of aged individuals [74,75,76] as well as those with Alzheimer’s [77,78,79,80], Parkinson’s [81,82], Huntington’s [83], and other neurological diseases [84]. With the desire to monitor lesion levels over time, measurements of 8-oxoG levels in the serum, urine, or cerebrospinal fluid (CSF) have also been explored as markers of oxidative stress in the brain. Significant elevation of 8-oxoG levels in the serum, urine, or cerebrospinal fluid from aging individuals or those with neurodegenerative disease or brain injury offers the opportunity for longitudinal monitoring of lesions during disease progression [85,95,103,104,105].

High-performance liquid chromatography (HPLC) and mass spectrometry (MS) are other longstanding techniques to measure 8-oxoG levels in the brain and other tissues of patients [78,85,97,98,99]. MS has also been used to measure elevated levels of 8-hydroxyadenine (8-OHA), 5-hydroxycytosine, thymine glycol, 5-hydroxyuracil, 4,6-diamino-5-formamido-pyrimidine (FapyAde), and 2,6-diamino-4-hydroxy-5-formamidopyrimidine of 2′-deoxyguanosine (FaPyGua) in various Alzheimer’s disease brain regions [78,86]. Other MS methods have been used to measure peroxidation and exposure-related adducts like acrolein-deoxyguanosine and *trans*-4-hydroxynonenal (HNE) with 2′-deoxyguanosine (HNE-dG) in the brain of Alzheimer’s patients [87,88]. Another MS method detects N^7^-guanine- 2-chloroethyl-ethyl-sulfide after mustard gas exposure in mice [89]. Increases in *O^6^*-methyldeoxyguanosine (*O^6^*-mG) DNA lesions were also measured after exposures to methylazoxymethanol (MAM), a metabolite of the cycad plant genotoxin cycasin [90]. Dietary or medicinal exposure to MAM is reportedly an etiological factor for Western Pacific amyotrophic lateral sclerosis and parkinsonism-dementia complex (ALS-PDC), a prototypical neurological disorder with neuropathological features of ALS, atypical parkinsonism, or an Alzheimer-like dementia [90].

As DNA adductomic techniques advance, they may identify additional DNA lesions in the brain of animal models, the human brain, and surrogate biological fluids linking endogenous and exogenous exposures to neurological diseases [106,107]. More than 200 chemicals are known to cause neurotoxicity, and many more are predicted to cause neurological disorders. The lack of specific antibodies for DNA lesions beyond 8-oxoG, 6,4-photoproducts, cyclobutane pyrimidine dimers, and thymine dimers, and the need to develop MS methods and isotopic dilution techniques for identification of specific DNA lesions in the brain currently limits our understanding of the implications of specific lesions in neurological disorders.

While not lesion-specific, a more precise assessment of DNA damage can be achieved by examining strand breaks and abasic sites using single-cell gel electrophoresis (Comet assay) [108]. Isolated nuclei from the parietal cortex and the caudate putamen of rats were shown to have elevated tail moments after the induction of ischemic injury [91,92]. Neurons and astrocytes isolated from aging rats also showed elevated tail moments by alkaline and neutral comet assays as a function of age. Modification of the comet assay to include enzyme treatment with 8-oxoguanine DNA glycosylase (OGG1) and uracil DNA glycosylase (UDG) showed a further increase in tail moments, confirming elevated oxidative and uracil lesions in aging brains [93]. Comet assays have also been used to assess genotoxic lesions in the brains of mice and rats from irradiation, chemotherapy, or other environmental exposures [87,88,94]. For human samples, comet DNA damage measurements typically occur in surrogate blood cell markers, like lymphoblasts, leukocytes, or buffy coat [95,96]. A compendium of comet protocols was recently published [108], and there are additional examples of applications of the comet assay to isolated brain cells or tissue to measure basal or induced DNA damage for biomonitoring or monitoring disease progression [91,92].

Lastly, DNA damage is commonly inferred in the brain by changes in DNA repair proteins. Mutations or loss of function in DNA repair proteins like aprataxin (APTX), tyrosyl-DNA phosphodiesterase 1 (TDP1), or ataxia telangiectasia mutated kinase (ATM) have well-known associations with neurodegenerative diseases. Next-generation whole tissue and single-cell sequencing efforts have focused on exploring the mutational landscape in neurological diseases. Mutational signatures identified by these efforts may offer insight into genotoxic exposures, like temozolomide, which induce DNA damage in the brain [109,110]. Additionally, many studies have characterized the gene and protein expression levels of DNA damage response and repair pathways. Key takeaways are diminished capacities for base excision repair through loss of OGG1, *O^6^*-methylguanine-DNA methyltransferase (MGMT), or other glycosylases [50,51]. Reduction in single-strand break repair and NHEJ proteins have also been measured, consistent with the high strand breaks measured by the abovementioned techniques [52,53]. Given the indirect nature of these characterizations on DNA damage levels, we refer the reader to many reviews detailing the alterations in DNA repair pathways and their potential implications for aging, neurodegeneration, and neuropsychiatric disease [8,9,23,111,112].

### 2.2. Sequencing-Based Methods

**Figure 1:**
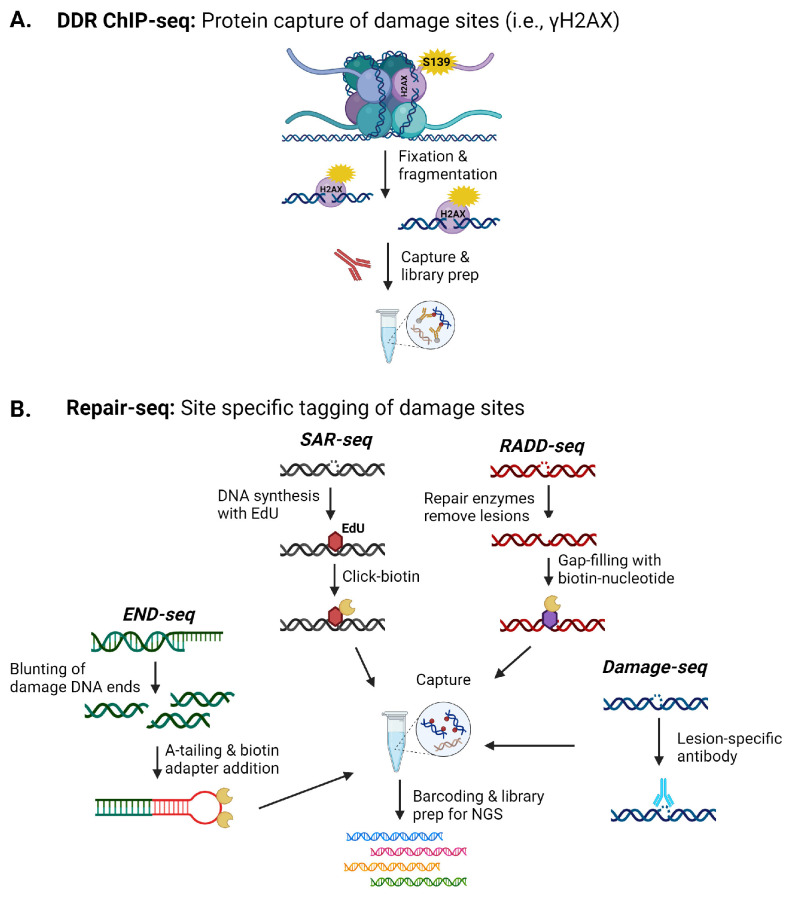
Examples of next-generation sequencing (NGS) techniques for detecting DNA damage within the brain. (**A**) Indirect sequencing of lesions occurs by capturing DNA damage response (DDR) proteins like γH2AX. (**B**) Various methods have been developed to directly capture DNA damage from DSB to base lesions for sequencing. Images were created with BioRender.

Beyond understanding the increased prevalence of specific DNA lesions, there is also substantial interest in mapping genomic sites more susceptible to these lesions and their resulting influence on gene expression, mutations, and translocations. Mapping of specific DNA damage sites has grown significantly over the past twenty years as sequencing technologies and our ability to isolate and enrich damaged sequences have improved. Several key strategies for isolating and sequencing DNA lesion or strand break sites within the genome involve lesion specific-antibody-based capture techniques, DNA repair protein-based capture strategies (modified chromatin immunoprecipitation (ChIP)-like), or the insertion of tagged or chemically modified nucleotides for enrichment (Figure 1) [102].

Several studies have used γH2AX-ChIP to pull down genomic sequences in the regions of DNA damage for analysis (Figure 1A). These studies mapped increases in DNA damage, inferred to be DSBs, within promoter regions of early response genes in stimulated neurons in response to fear learning [1]. Applying this method to the prefrontal cortex and hippocampal regions also identified increased DSB induced by contextual fear conditioning across these regions, with clusters of damage observed in genes associated with synaptic processes, early response genes, RNA-binding genes, and cytoskeleton-related genes [2].

Given the propagation of the γH2AX signal, to improve resolution at the site of the strand break, END-seq and synthesis associated with repair sequencing (SAR-seq) were recently used to examine DNA damage in post-mitotic neurons (Figure 1B) [100]. END-seq, which directly ligates a sequencing adaptor to the ends of strand breaks, and S1 END-seq, a modification of END-seq where recombinant single-strand-specific S1 nuclease is used to convert SSBs into DSBs to allow detection of SSBs by END-seq were used to isolate DNA damage events from DNA repair events [100]. SAR-seq incorporates EdU, which is then biotinylated to allow isolation and high-throughput sequencing [100]. Active synthesis in post-mitotic neurons will be associated with DNA repair, though the synthesis length must be sufficient for labeling for this technique to work. These techniques were applied to induced pluripotent stem cell-derived neurons and primary neurons. Using these sequencing techniques, SSBs with active DNA synthesis were observed in neuronal enhancer regions associated with cytosine demethylation events and coordinated long-patch SSB repair. DSBs were not associated with the SSBs at neuronal enhancers [100].

Currently, only these DNA damage sequencing methods have been reported for mapping DNA strand breaks within the brain. More than 40 DNA damage sequencing techniques have been reported for detecting specific lesions, i.e., 8-oxoG, platinated crosslinks, pyrimidine dimers, photoproducts, benzo[a]pyrene derivatives, abasic sites, ribonucleotides, and uracils, and SSB and DSBs (Figure 1B) [101,102]. An obvious limitation to the widespread application of these methods is the amount of fresh material needed for DNA isolation and the specific antibodies needed for lesion enrichment, which also limits the identification of specific lesion types. As the DNA damaging event becomes rarer, the amount of tissue or cells needed for enrichment increases. However, the successful use of γH2AX-ChIP, END-seq, and synthesis associated with repair sequencing (SAR-seq) to primary cells and isolated brain regions suggests techniques that incorporate covalent labeling of DNA sites by Click chemistries or biotinylation, i.e., SAR-seq, Breaks Labeling, Enrichment on Streptavidin, and Sequencing (BLESS), breaks labeling in situ and sequencing (BLISS), or Repair Assisted Damage Detection sequencing (RADD-seq), can be employed to map a number of lesions within various neural cell types or specific brain regions (Figure 1B) [101,102].

One additional developing area is the application of nanopore sequencing to identify DNA modifications or adducts in isolated DNA from brain cells or regions. While no peer-reviewed manuscripts have described the application of nanopore sequencing to DNA damage in the brain, a recent preprint described using nanopore sequencing to examine structural variants and methylation sites within DNA isolated from the frontal cortex of Alzheimer’s patients [113]. While adductomic signatures are still being developed for nanopore sequencing reads, as this technology is refined, it will likely shed new light on the mixtures of DNA damage accumulated within the brain and neural cells during genotoxic exposures, trauma, aging, or neurological disorders.

### 2.3. Fluorescent Reporters for DNA Damage

There has been significant emphasis on detecting and measuring DNA strand breaks, given their prevalence in neurodegeneration, neuropsychiatric disorders, and after neurotrauma. While some driving events for these strand breaks have been discovered in memory, learning, and transcriptional regulation, the roles of other events, like genotoxic exposures, are still unclear. Similar to cancer, genotoxic exposures over time could lead to genomic instability, driving mutations within the brain that lead to sporadic neurodegeneration. Genotoxic exposures may also induce transcriptional mutagenesis (TM) through DNA damage, leading to mutant proteins. MAM and Methylnitronitrosoguanidine (MNNG) have recently been shown to induce TM in mouse primary neurons [114]. Environmental exposure may also alter critical antioxidant mechanisms, promoting elevated levels of oxidative stress and leading to strand breaks and dysregulation of DNA repair pathways, i.e., paraquat exposure in Parkinson’s disease [2,115]. Hyperglycemia, diabetes, and inflammatory conditions can promote advanced glycation end products, like methylglyoxal or aldehyde species, leading to elevated strand breaks and cognitive decline [116]. To understand the role of genotoxic exposures, more real-time DNA or longitudinal DNA damage measurements are needed to link damaging events to disease etiology.

**Figure 2:**
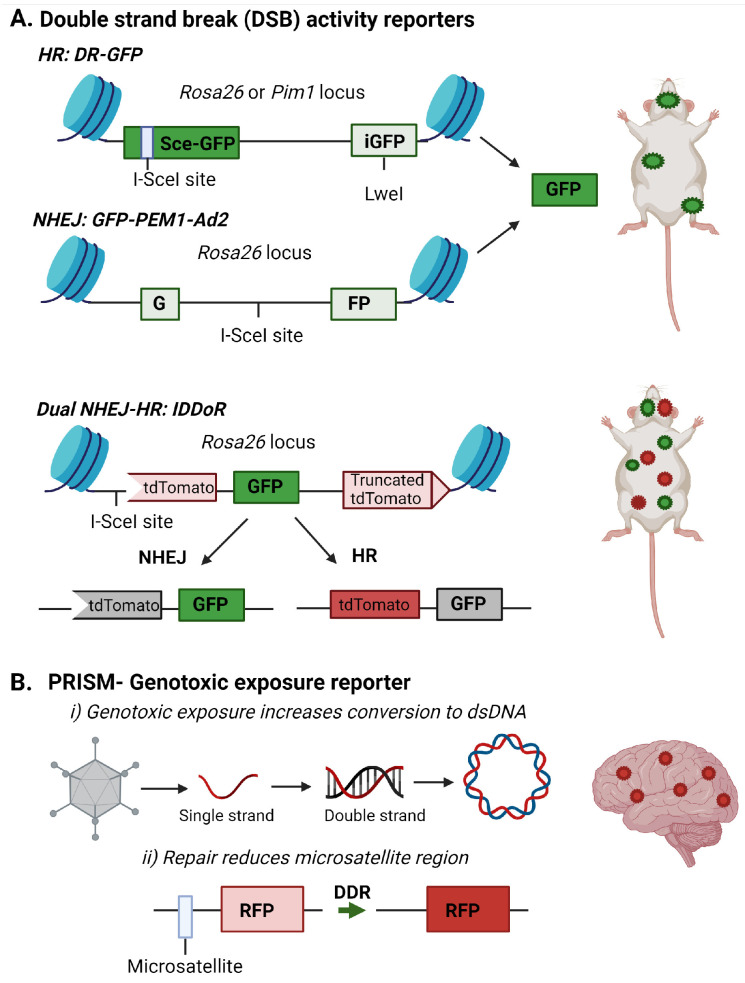
Fluorescent reporter detecting DNA repair or genotoxic exposures in the brain. (**A**) Fluorescent reporters are incorporated into specific loci within the mouse genome to allow DNA repair of double-strand breaks (DSB) by non-homologous end joining (NHEJ) or homologous recombination (HR) to be monitored. Images are adapted from [54,56,57]. (**B**) The PRISM reporter detects DNA damage induced by genotoxic exposures in the brain. The RFP reporter is incorporated by adenoassociated and uses two methods to detect DNA damage response and repair in the brain. The first is the conversion of a single strand genome to a double strand genome, and the second is the alteration of the microsatellite repeat to allow RFP transcription. This image is adapted from [58]. Images were created with BioRender.

Fluorescent reporters offer unique methods for tracking DNA damage and repair in vivo. Over the past 20 years, highly advanced fluorescent reporter systems have been developed to monitor DNA damage response and repair. Based on host cell reactivation assays, plasmid and gene cassettes have been designed to incorporate known DNA lesions or break sites into fluorescent genes (Figure 2). DNA repair removes the transcription-blocking damage or restores the fluorescent gene coding sequence to allow detection of the repair event through the expression of the reporter gene [117,118,119]. These assays indirectly infer DNA damage presence by measuring the capacity or deficiency of specific DNA repair pathways. For example, loss of MGMT expression, frequently observed in glioblastoma, would result in elevation of *O^6^*-mG and lack of reactivation of a reporter gene containing this lesion [120]. Similarly, defects in NHEJ or HR could be detected through a lack of restoration of fluorescent protein expression in substrate targeting these repair pathways (Figure 2A) [118,119].

These assays are most amenable to primary or immortalized cells since the lesions often need to be incorporated into the reporter before introduction into the target cells. However, several groups have created mouse models to measure NHEJ or HR efficiency in vivo in brain, liver, mammary, bone marrow, and intestinal tissues (Figure 2A) [54,55,56,121]. Relevant to measuring DSB repair in the brain is the recent creation of an IDDoR (inducible dual-fluorescence-based double-strand break repair reporter) mouse, which allows the simultaneous measurements of NHEJ and HR through GFP and tdTomato reporters, respectively, incorporated into the Rosa26 locus of all tissues in the mouse (Figure 2A) [57]. The mouse also contains an inducible I-SceI to produce the break site in the reporter for assessing repair. The study validated the expression of I-SceI and the reporters in the kidney, brain, heart, intestine, pancreas, stomach, and skin of the IDDoR mouse [57].

The need to induce the strand break with I-SceI limits the ability of these reporters to monitor induced DNA damage events. To address the need to monitor endogenous or genotoxic strand breaks in vivo, an additional reporter system, Probe with a viRal proxy for the Instability of DNA surveillance/repair in Somatic brain Mosaicism (PRISM), has been designed to target neurons and report induced genomic instability. This system first exploits the property that DNA damage enhances the uptake of DNA or RNA constructs and viral vectors [122,123]. In this case, a single-strand recombinant adenoassociated virus (rAAV) contains an error-prone microsatellite repeat between the start codon and the protein-coding sequence of Cre recombinase or fluorescent proteins, creating a frameshift mutation preventing the synthesis of the functional protein. Microsatellite instability was previously used to regulate fluorescent protein expression to achieve sparse fluorescent labeling of neurons, and Mosaicism with Repeat Frameshift [124]. However, this strategy was not specifically used to examine genomic instability.

Under genotoxic stress, the DNA damage machinery, which typically prevents rAAV genome processing, is used by the cell for repair. This lack of viral defense allows the rAAV reporter to be processed to double-strand DNA for gene expression. The ongoing DNA damage response in the cell can alter the error-prone microsatellite increase, inducing the expression of the reporter gene [58]. Using the Cre-mediated tdTomato reporter mouse, the authors demonstrated genotoxic stress and altered DNA repair processes could be detected in the brain in vivo after exposure to genotoxins like paraquat and doxorubicin (Figure 2B) [58].

The desire to dynamically monitor DNA repair events in vitro and in vivo has led to significant advancements in reactivation assays and reporter system designs. While these methods do not specifically measure elevated lesions in the brain, they offer strategies for assessing exposures and initiation of DNA repair processes that are more functionally oriented than inferred from protein expression levels.

Additionally, they offer the ability to monitor DNA damage and repair in live animals and allow for high-resolution analysis of fluorescent signals in cells and tissues isolated from these engineered or exposed animals [54,55,56,57]. While the current generation of these probes uses standard fluorescent proteins, their designs and targeted incorporation into brain cells could easily be enhanced to allow the application of in vivo and in vitro super-resolution imaging techniques [125,126,127]. Super-resolution microscopy methods applied in the neurosciences have provided insight into cellular communication, signaling, and structure [125,126,127]. While super-resolution techniques have not yet been applied to the investigation of DNA lesions in the brain, they are already being used in cell models to examine DNA damage and repair [128,129,130]. As with sequencing methods, the rapid improvement in these methods and their novel adaptions to brain cells will likely see their incorporation into assessments of DNA damage and repair in aging, neurodegeneration, and neuropsychiatry in the near future.

### 2.4. Mitochondrial DNA Damage

In addition to nuclear DNA damage, the mitochondrial genome is susceptible to DNA damage from endogenous and exogenous sources. If mitophagy does not remove damaged mitochondrial DNA (mtDNA), it can impact mitochondrial function and cellular energetics. Given the high energetic demands of the brain, it is not surprising that DNA damage within the mitochondria and genetic mutations in mtDNA are associated with aging, neurodegeneration, and cognitive impairment [9,59,60].

Measuring DNA damage specifically within the mtDNA has proved challenging because classic methods for quantifying DNA lesion levels, from immunoassay to mass spectrometry, require considerable amounts of purified mtDNA. While not impossible to achieve, the isolation of mtDNA from limited cell and human samples has made this approach less feasible. Whole brains from Alzheimer’s patients were used to quantify elevated 8-oxoG in mtDNA by HPLC in 1994 [97]. Therefore, long-range PCR is the most common method for assessing mtDNA lesions [131]. This approach assumes that DNA lesions or strand breaks within the mtDNA limit the processivity of DNA polymerases and reduce the accumulation of long PCR substrates (~10–12 Kbp). The level of DNA damage is then inversely proportional to the amount of long PCR product accumulated [132]. A similar long-PCR strategy can also be applied to assess nuclear DNA damage since isolated DNA samples from neural tissues would contain both genomes [132,133]. Then, the relative DNA damage levels between the two genomes can be compared.

qPCR or long-range PCR methods have been applied to measure DNA damage within the brain and in surrogate cells such as buffy coats or PBMCs. Elevated levels of mtDNA damage, including deletions, have also been reported for Parkinson’s, Alzheimer’s, Huntington’s, and acute brain injury [59,61,62,63,64,65]. In Parkinson’s patients’ brains, differences in mtDNA were observed between midbrain and cortical neurons after rotenone exposure [61]. Elevated levels of mtDNA damage were also observed in mouse midbrain and cortex after exposure to rotenone. Additionally, treatment of isolated DNA with formamidopyrimidine DNA glycosylase (FPG) before qPCR further reduced mtDNA amplification, confirming the presence of oxidative DNA lesions within the mtDNA [66]. They also identified the D-loop of the mitochondrial genome as being the most susceptible to H_2_O_2_-induced damage in the brain [66].

Blood-based mtDNA analysis from cell-free mtDNA or in the buffy coat showed elevated mtDNA damage and deletions in individuals with Alzheimer’s and Parkinson’s disease and traumatic brain injury [67,68,69]. A recent report suggested that mtDNA damage analysis in blood cells may be a suitable candidate biomarker for Parkinson’s disease with the potential for early disease detection [71]. Similar suggestions for the predictive value of mtDNA damage or copy number analysis have been made for Alzheimer’s disease and multiple sclerosis [67,70].

A limitation of this approach is that the specific DNA lesions cannot be identified. However, the class of DNA lesion was confirmed by Gureev et al. by adding FPG to their mtDNA assays, and similar approaches could be used for detecting other lesion types [66]. The qPCR approach also allows specific amplicons or regions to be examined for their susceptibility to DNA damaging agents without sequencing [66].

### 2.5. Enzyme-Mediated DNA Damage Detection Assay

A final emerging technique for assessing DNA damage in the brain takes advantage of the recognition of DNA lesions by DNA repair enzymes. DNA repair enzymes have already evolved to recognize and excise DNA lesions. Their enzymatic reactions with damaged DNA and the end products they leave within the DNA backbone are well described. This knowledge offers the unique opportunity to use permissive DNA polymerases to tag excised lesion sites with modified nucleic acids for downstream detection.

These techniques are more commonly used on isolated DNA, i.e., pyrimidine dimer glycosylase (PDG) removes ultraviolet light (UV)-induced DNA adducts and DNA polymerase I tags the lesion site with a fluorescently labeled dNTPs [134]. Additional examples for isolated DNA include the use of enzymatic cocktails of DNA repair enzymes containing bacterial DNA glycosylases, endonuclease IV (EndoIV), endonuclease VIII (EndoVIII), FPG, T4 PDG, uracil DNA glycosylase (UDG), and *Bst* DNA polymerase, to measure DNA damage induced by several environmental agents [135,136,137,138].

These techniques could be applied to isolated DNA from aging brains, diseased brains, or brains exposed to environmental agents, similar to the MS and immunoassay methods described above. The benefit of using this methodology instead of the more precise quantitation by MS is the requirements for much smaller amounts of material (nanogram to microgram) and the broader spectrum detection of lesions without the need for specific reference lesions for mass fragmentation and quantification analysis. The advantage over immunoassays is that there is no requirement for antibodies specific to different lesions. Broad classes of lesions can be detected based on the DNA repair enzymes used. EndoIV recognizes abasic sites, EndoVIII recognizes thymine glycol, 6-hydroxy-5,6-dihydrothymine, 5, 6-dihydroxythymine, 5-hydroxy-5-methylhydanton, uracil glycol, urea, and methyltartronylurea. FPG detects fapy-guanine, methy-fapy-guanine, aflatoxin Bl-fapy-guanine, 7,S-dihydro-S-oxoguanine (S-oxoguanine), 8-oxoadenine, fapy-adenine, 5-hydroxy-cytosine, and 5-hydroxy-uracil. T4 PDG recognizes cyclobutane pyrimidine dimers, 6-4 photoproducts, and abasic sites. UDG recognizes uracils and oxidation products of cytosine. These enzymes can be used individually, similar to the COMET and qPCR methods described above, or simultaneously to assess DNA lesions within isolated DNA [66,93]. An additional benefit to enzyme-mediated detection is that the tagged DNA can also be captured and enriched for next-generation sequencing techniques that examine the genetic location of DNA damage, as described in Section 2.2 [101]. These techniques would also be compatible with isolated DNA from blood or other fluids containing cells or cell-free DNA.

**Figure 3:**
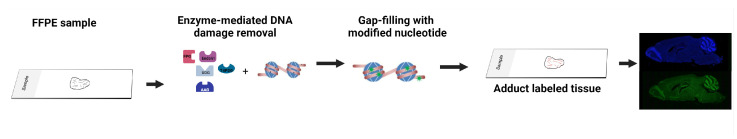
Overview of the enzyme-mediated strategies for detecting DNA lesions in formalin-fixed paraffin-embedded tissues. Images were created with BioRender.

Beyond isolated DNA, the Repair Assisted Damage Detection (RADD) enzyme-mediated DNA damage detection strategy has optimized the detection of DNA damage within cells and tissues (Figure 3) [139,140,141]. The technique has also been adapted to next-generation sequencing of the genomic location of DNA damage (RADD-seq) [101]. RADD uses a cocktail of the bacterial DNA repair enzymes described above (3-alkyladenine DNA glycosylase (AAG), FPG, T4 PDG, UDG, Endo VIII, and Endo IV) to detect a broad spectrum of DNA lesions and even DNA strand breaks within the nuclei of cells or across heterogeneous tissues [139,140,141,142,143]. Individual lesion cocktails focused on specific lesions have been used in ovarian and prostate tissues to examine the DNA lesion heterogeneity and colocalization with protein markers of interest [142,143]. Of note, oxRADD, a cocktail of FPG, Endo IV, and Endo VIII, was used to detect differences in DNA lesions between African American and European American prostate cancer tumors and multiplexed with PD-L1 expression in ovarian tumor samples [142,143].

Given the technique has not yet been applied to brain tissue, we optimized the application of RADD to the brains of female wild-type C57BL/6 fed standard rodent chow or chow-supplemented with 0.03127 mg of trans-resveratrol (RSV) per g of chow (Supplementary Methods). RSV is a polyphenol that protects against oxidative DNA damage and reduces DSBs [144]. We applied RADD to determine if a reduction in DNA damage could be observed within the brain.

**Figure 4:**
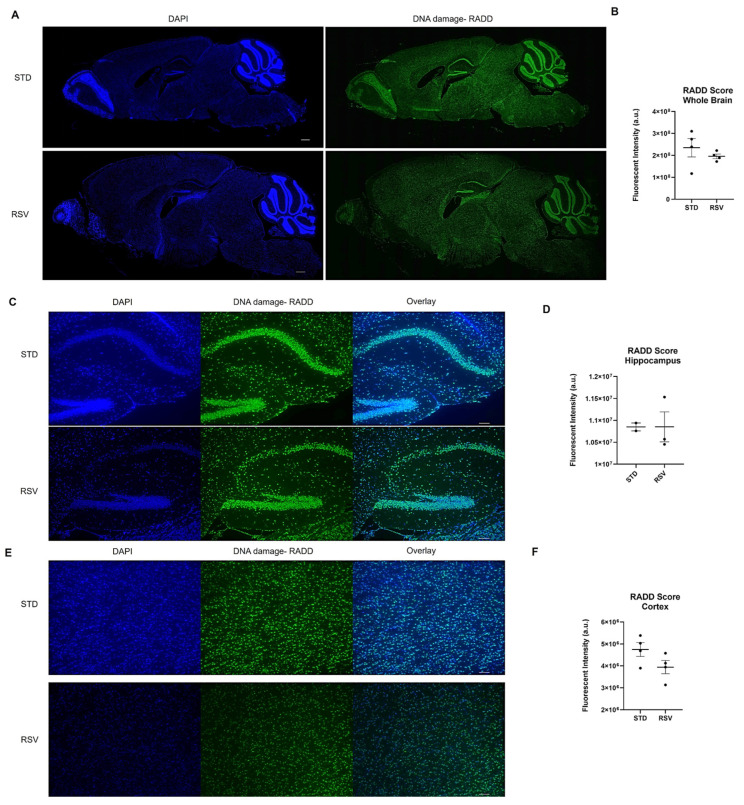
DNA damage analysis on mouse brain from 10-month-old wild-type C57BL/6 fed standard chow (STD) or resveratrol (RSV) containing chow for 2 months. (**A**) Representative stitched image of the entire 5 µm section imaged at 10× from each mouse brain where DNA damage analysis by RADD was performed. The scale bar is 500 µm. (**B**) Mean fluorescence intensity ± standard error of the mean (SEM) of the RADD signal for the brain with the cerebellum and olfactory bulb excluded (*n* = 4 STD and RSV). (**C**) Individual images at 10× of STD and RSV-fed mice hippocampus. (**D**) Mean fluorescence intensity ± SEM of the RADD signal in the hippocampus for a subset of mice. (**E**) Individual image at 10× of STD and RSV-fed mice cortex. (**F**) Mean fluorescence intensity ± SEM of the RADD signal in the cortex for a subset of mice. Scale bar = 100 µm. Detailed methods are in the Supplementary Materials.

Mice were maintained on the standard (STD) diet until 8 months of age, then randomized into the two diet groups. The mice were then fed STD or RSV diets for two months and then sacrificed as described in [145]. Formalin-fixed paraffin-embedded hemi-brains were sectioned at 5 µm, and we performed the RADD assay for the broad spectrum of DNA lesions, oxidative, crosslinks, uracils, abasic sites, and alkylation (Supplementary Methods). Figure 4 shows the entire brain section stained by the RADD assay and the RADD score, excluding the cerebellum and olfactory bulb. RSV reduced the broad spectrum of DNA damage measured by RADD in the whole brain. A limited analysis of the DNA damage intensity between the cortex and hippocampal regions is also shown in Figure 4, revealing a reduction of DNA damage in the cortex but not the hippocampus. While the limited sample number reduces the ability to draw significant conclusions, the results here are consistent with previous reports demonstrating RSV reduces radicals, up-regulates antioxidant-related genes, and activates sirtuin 1 (SIRT) to provide neuroprotective effects against ischemia and neurodegenerative diseases [146,147,148].

**Figure 5:**
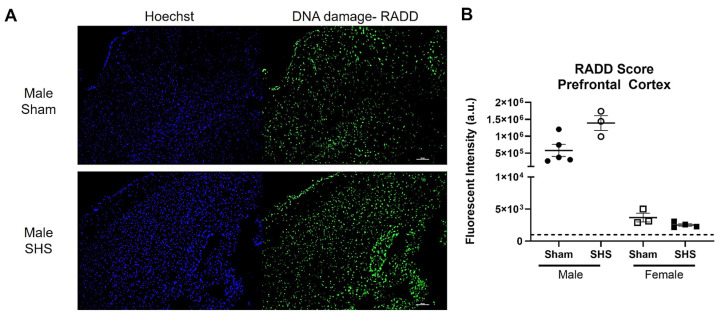
RADD DNA damage analysis of the brain from sham or chronic second-hand smoke (SHS) exposed C57BL/6 mice. (**A**) Representative 10× images of the DNA damage analysis performed on the prefrontal cortex of male mice. (**B**) Mean fluorescence intensity ± SEM of the RADD signal measured within three different sections of the prefrontal cortex from male and female mice. The scale bar is 100 µm.

We also performed a broad-spectrum DNA damage analysis by RADD on freshly frozen brain sections from sham or chronic second-hand-smoke (SHS)-exposed C57BL/6 mice [149]. Previous immunohistochemical studies have shown that oxidative DNA damage is elevated, and DNA repair is altered in the brains of SHS-exposed mice [150]. A single brain section (20 µm) was analyzed for each group and sex. We observed increased DNA damage in the SHS-exposed male prefrontal cortex compared to the sham treatment (Figure 5). The female mouse brains showed no difference in DNA damage between sham and SHS exposure. The RADD results are consistent with previous reports showing sex-specific differences in DNA damage levels after SHS exposure [149]. The RADD damage levels are also consistent with the elevated levels of 8-oxoG observed in the prefrontal cortex of SHS-exposed mice [150]. Lower protein expression of Ogg1 and elevated Ape1 were also observed in the prefrontal cortex of SHS-exposed mice [150,151]. Elevated levels of 8-oxoG were also found in the hippocampal regions CA1, CA3, and CA4 of SHS-exposed mice, with the increasing oxidative damage negatively correlated with cognitive performance [151]. In the hippocampal neurons, Ape1 levels were increased, but there were fluctuations in the expression of Ogg1, with increased levels observed in CA1 and CA4 and reduced levels in CA3 [150]. These results demonstrate the importance of measuring DNA lesions because levels of DNA repair proteins do not always correlate with repair capacity [139,150,151,152].

Together, these applications demonstrate that DNA adduct analysis can be carried out with RADD on brain sections with limited modifications to the original protocol [140]. Additionally, the RADD protocol does not limit multiplexing of the RADD signal with cell markers or DNA repair proteins of interest, so a spatial analysis of the DNA damage within different brain regions and specific neural cells could be conducted using this method simultaneously.

## 3. Conclusions

DNA damage, whether arising from endogenous or exogenous sources, appears to play a significant role in aging, neurodegeneration, neurotrauma, and neuropsychiatric disorders. While numerous studies have examined DNA damage within the brain, the most frequently used methods only indirectly measure DNA damage through DNA damage signaling by γH2AX or are limited to analysis of specific lesions (i.e., 8-oxoG). Application of mass spectrometry has revealed that aldehyde and acrolein DNA adducts occur in the neurodegenerative-diseased brain, suggesting that our understanding of the role of exogenous and endogenous exposures is limited by the most commonly used methods for assessing DNA damage in the brain. Here, we have reviewed several emerging methods for measuring DNA damage and specific DNA lesions within the brain, which will improve our understanding of genotoxic exposures, genomic susceptibility, and the microenvironment or cell type that influences DNA damage.

While applying these techniques to the brain has lagged behind those in cancer and other diseases, the unique insight they can provide about drivers of neurological disease will drive their adoption in the near future. Advancements in sequencing methods will provide more information about genomic susceptibility to DNA damage in the brain and may offer insight into subsets of mutational patterns that could inform disease etiology. Fluorescent reporters, super-resolution imaging, and spatial imaging techniques will offer crucial insights into disease mechanisms, cellular susceptibility, and potential therapeutic targets. Additionally, the ability to precisely and quantitatively map DNA damage accumulation over time, after traumatic events, or after environmental exposures will provide new strategies for evaluating interventions, treatments, and/or mitigation strategies to prevent, delay, or reverse the onset of debilitating symptoms. With the aging population and rising rates of neurological diseases, advanced methods and technologies are critically needed for early diagnosis, progression monitoring, and therapeutic development and testing to improve quality of life and reduce the economic and emotional burdens of neurological diseases.

## Data Availability

All data are included in this manuscript.

## Supplementary Materials

The following supporting information can be downloaded at: https://www.mdpi.com/article/10.3390/ijms25137021/s1.
