# Supplementary material for: Novel Techniques for Mapping DNA Damage and Repair in the Brain
Source: Int J Mol Sci. 2024 Jun 27;25(13):7021. doi: 10.3390/ijms25137021 (PMC11241736; doi:10.3390/ijms25137021)
Supplement: Supplementary file 1 [file ijms-25-07021-s001.zip › ijms-3058247-supplementary.pdf]

Supplemental Methods

Repair Assisted Damage Detection (RADD) in C57BL/6 mice fed an antioxidant diet

Dr. Joanne Allard provided a subset of mouse brains from wild type C57BL/6 from a study examining the effects of a resveratrol diet. Mice were maintained on standard chow (Diet no. D11112201, Research Diets, New Brunswick, NJ, USA) until 8 months of age. The mice were then randomized into two diet groups, standard chow (STD) and chow containing 0.03127 mg of trans-resveratrol (RSV) per gram of chow, and diet was maintained for two months. Mice were sacrificed, and their brains were collected as described in *(Mulgrave, Alsayegh et al. 2023).* Mouse hemi brains from STD and RSV-fed mice were provided frozen in OCT. Brains were thawed and paraffin-embedded by the Comparative Pathology Laboratory at the University of Alabama at Birmingham. The paraffin-embedded hemi brains were cut into 5 µm sections.

Slides were placed on a heat block set at 65°C and incubated for 10 min to melt the paraffin. Slides were then placed directly in 100% xylene (Fisher Scientific, Waltham, MA, USA, X3P) and incubated three times for 10 min each. Slides were rehydrated to water through sequential incubations in ethanol and water mixtures. Specifically, slides were incubated for 5 min each in sequential order of 100% ethanol (VWR, 89125-170)-0% water; 95% ethanol-5% water; 70% ethanol-30% water; 50% ethanol-50% water; 0% ethanol-100% water. Rehydrated slides were then placed in plastic Coplin jars with 50 mL of 10 mM sodium citrate pH 6.0 (VWR, JT3646-1), 1 mM EDTA pH 8.0 (Thermo Scientific, Waltham, MA, USA, AM9260G), and 0.05% Tween 20 (Thermo Scientific, AAJ20605AP) in water and microwaved three times for 18, 15, and 15 secs with 5 min rest periods between for antigen retrieval. The temperature of the solution was microwaved to reach 90 °C and was maintained at this temperature for ~ 20 min with the additional microwave cycles. Slides were cooled with five changes of water. Slides were then placed in phosphate-buffered saline (PBS, Hyclone, Logan, UT, USA, SH30028FS) with 0.5% triton X-100 (Sigma Aldrich, T8787) and 0.5% Tween 20 and incubated for 1 h at 37°C. The slides were then washed three times with PBS. Slides were briefly dried, and the tissues were outlined with a PAP pen.

For broad-spectrum DNA damage detection (RADD), all the enzymes in Table 1, UDG, FPG, T4PDG, AAG, EndoVIII, and EndoIV, were added to the tissues and incubated for 1 h at 37 °C. The gap-filling solution (Table 1) was added directly to the lesion removal solution and incubated for 1 h at 37 °C. Slides were then washed three times in tris-buffered saline (TBS, MP biomedicals, Solon, OH, USA, 08W00020) with 0.1% Tween 20 for 5 min each and blocked in 2% BSA (Jackson Immuno, West Grove, PA, USA, 001-000-162) in PBS for 30 min at room temperature (RT, ~24 °C). Anti-digoxigenin (Dig) antibody (Abcam, Cambridge, UK, #ab420 clone 21H8) was incubated at a dilution of 1:250 in 2% BSA in PBS at 4 °C overnight.

The next day, slides were washed three times in PBS for 5 min each, and Alexa Fluor 546 goat anti-mouse secondary (Life Technologies, Carlsbad, CA, USA, A21235) was incubated at a dilution of 1:400 in 2% BSA in PBS for 1 h at RT. DAPI (Life Technologies, Carlsbad, CA, USA, D1306) was added at a final dilution of 1:800 for 15 min at RT to stain the nuclei. Slides were washed three times in PBS for 5 min each, briefly dried, and mounted with coverslips using ProLong Gold Antifade reagent (Life Technologies, Carlsbad, CA, USA, P36930). Slides were allowed to dry overnight in the dark at RT and visualized using a Keyence BZ-X800 microscope or stored at 4 °C until analysis.

Images were acquired using a Plan-Apochromat 10x/0.45 objective. Images were acquired using the stitch edges settings, with the edges of the tissue defining the imaging area. Entire tissues required 80-150 individual images to fully cover the tissue area, with each image acquired at 920x760 resolution. Final sections were stitched using the Keyence BX Analyzer

software, and a large TIFF of the entire image at the original resolution was exported for analysis.

TIFF images were imported into the Nikon NIS Elements software to create an ND file. A binary threshold was applied to each image, and specific regions of interest (ROI) were drawn using the bezier function. The fluorescence intensity for each ROI was exported for analysis. The final mean fluorescent intensity for all brain ROIs analyzed is reported in arbitrary units ± standard error of the mean. Data were plotted using GraphPad Prism software. Significance was examined using a Student's t-test, but none of the results reached a p-value < 0.05.

Table 1. RADD reaction conditions. RADD is performed in two sequential reactions without aspirating reagents between reactions. The lesion processing mix (left) is placed on prepared tissues and placed in a humidified incubator. The gap-filling mix (right) is added directly to the lesion processing mix and incubated for an additional hour. The reagents are then aspirated, and the cells are washed and incubated with an anti-digoxigenin antibody.

**Full RADD Lesion Processing Mix**	**Per 100 µL Reaction Volume**	**Gap-Filling Mix**	**Per 100 µL Reaction Volume**
UDG (NEB M0280)	2.5 U	Klenow exo^{-} (Thermo Fisher, Waltham, MA, USA, EP0422)	1
FPG (NEB M0240)	4 U	Digoxigenin dUTP (Sigma Aldrich, St. Louis, MO, USA, 11093088910)	0.1
T4 PDG (NEB M0308)	5 U	Thermo Pol Buffer (NEB B9004)	10 µL
EndoIV (NEB M0304)	5 U		
AAG (NEB M0313)	5 U		
EndoVIII (NEB M0299)	5 U		
NAD^+ (100x, NEB B9007)	500 µM		
BSA (Sigma Aldrich, St. Louis, MO, USA)	200 µg/mL		
Thermo Pol Buffer (NEB B9004)	10 µL		

Repair Assisted Damage Detection (RADD) in C57BL/6 sham and second hand smoke (SHS) exposed mice

Dr. Glen Kisby provided frozen tissue sections adhered to a functionalized cover slide for analysis from the animal study described in (Raber, Perez et al. 2021). The fresh frozen tissue was heated briefly at 65°C to adhere it to the slide. The tissue was then placed in the plastic Coplin jars with 50 mL of 10 mM sodium citrate pH 6.0, 1 mM EDTA pH 8.0, and 0.05% Tween 20 in water and microwaved three times for 18, 15, and 13 secs with 5 min rest periods between for antigen retrieval. The temperature of the solution was microwaved to reach 90 °C and was maintained at this temperature for ~ 20 min with the additional microwave cycles. Slides were

cooled with five changes of water. Slides were then placed in phosphate-buffered saline (PBS, Hyclone, Logan, UT, USA, SH30028FS) with 0.5% triton X-100 (Sigma Aldrich, T8787) and 0.5% Tween 20 and incubated for 20 min at 37°C. The slides were then washed three times with PBS. Slides were briefly dried, and the tissues were outlined with a PAP pen.

RADD analysis was conducted as described above, with the only difference being the application of Hoescht instead of DAPI. Imaging and analysis were also conducted as described above.

Mulgrave, V. E., A. A. Alsayegh, A. Jaldi, D. T. Omire-Mayor, N. James, O. Ntekim, E. Walters, E. O. Akala and J. S. Allard (2023). "Exercise modulates APOE expression in brain cortex of female APOE3 and APOE4 targeted replacement mice." Neuropeptides **97**: 102307.

Raber, J., R. Perez, E. R. S. Torres, D. Krenik, S. Boutros, E. Patel, A. C. Chlebowski, E. R. Torres, Z. Perveen, A. Penn, D. B. Paulsen, M. G. Bartlett, E. Jia, S. Holden, R. Hall, J. Morre, C. Wong, E. Ho, J. Choi, J. F. Stevens, A. Noel, G. Bobe and G. Kisby (2021). "Effects of Chronic Secondhand Smoke (SHS) Exposure on Cognitive Performance and Metabolic Pathways in the Hippocampus of Wild-Type and Human Tau Mice." Environ Health Perspect **129**(5): 57009.
